# Design of Nickel Supported Hierarchical ZSM-5/USY Zeolite Bifunctional Catalysts for One-Pot Menthol Synthesis via Liquid-Phase Citral Hydrogenation

**DOI:** 10.3390/molecules28020743

**Published:** 2023-01-11

**Authors:** Abdul Karim Shah, Ghulam Taswar Shah, Aqeel Ahmed Shah, Yeung Ho Park, Ayaz Ali Shah, Mooseok Choi, Shoaib Ahmed, Syed Nizamuddin Shah Bukhari, Ali Dad Chandio, Muhammad Atta Mahar, Muhammad Ali Shar, Abdulaziz Alhazaa

**Affiliations:** 1 Department of Chemical Engineering, Dawood University of Engineering and Technology, Karachi 74800, Pakistan; 2 Fine Chemical Process Laboratory, Department of Chemical Engineering, Hanyang University, Sangnok-su, Ansan 15588, Republic of Korea; 3 Wet Chemistry Laboratory, Department of Metallurgical Engineering, NED University of Engineering and Technology, University Road, Karachi 75720, Pakistan; 4 Department of Energy and Environment Engineering, Dawood University of Engineering and Technology, Karachi 74800, Pakistan; 5 Department of Basic Science, Dawood University of Engineering and Technology, Karachi 74800, Pakistan; 6 Department of Mechanical and Energy Systems Engineering, Faculty of Engineering and Informatics, University of Bradford, Bradford BD7 1DP, UK; 7 Department of Physics and Astronomy, College of Science, King Saud University, Riyadh 11451, Saudi Arabia

**Keywords:** citral to menthol, Ni-H-ZSM-5, mesopores, acidity, catalytic activity

## Abstract

Nickel-supported hierarchical zeolite catalysts were prepared through a desilication reassembly process under optimized conditions and applied in one-pot menthol synthesis. In this work, the hierarchical zeolite-supported metal bifunctional catalysts were prepared with the help of desilication re-assembly and wetness impregnation techniques and applied in menthol synthesis via citral hydrogenation. The prepared catalysts were characterized using PXRD, BET, FE-TEM, NH_3_-TPD, H_2_-TPR, pyridine adsorption, and ICP-OES techniques. As a result, the physicochemical and acidic properties, such as mesopore surface area, metal dispersion, acidity, catalytic activity, and strong Lewis acid sites of pure microporous ZSM-5/USY zeolites, were significantly improved. Consequently, with the occurrence of superior physicochemical and acidic properties, the Ni/HZ-0.5 M catalyst exhibited outstanding catalytic activity (100% conversion, TOF 7.12 h^−1^) and menthol selectivity (83%, 4 h) with uniform stability at 100 °C, 1.0 MPa hydrogen. Similarly, the cracking rate decreased with the decrease in Bronsted acid sites.

## 1. Introduction

**Scheme 1:**
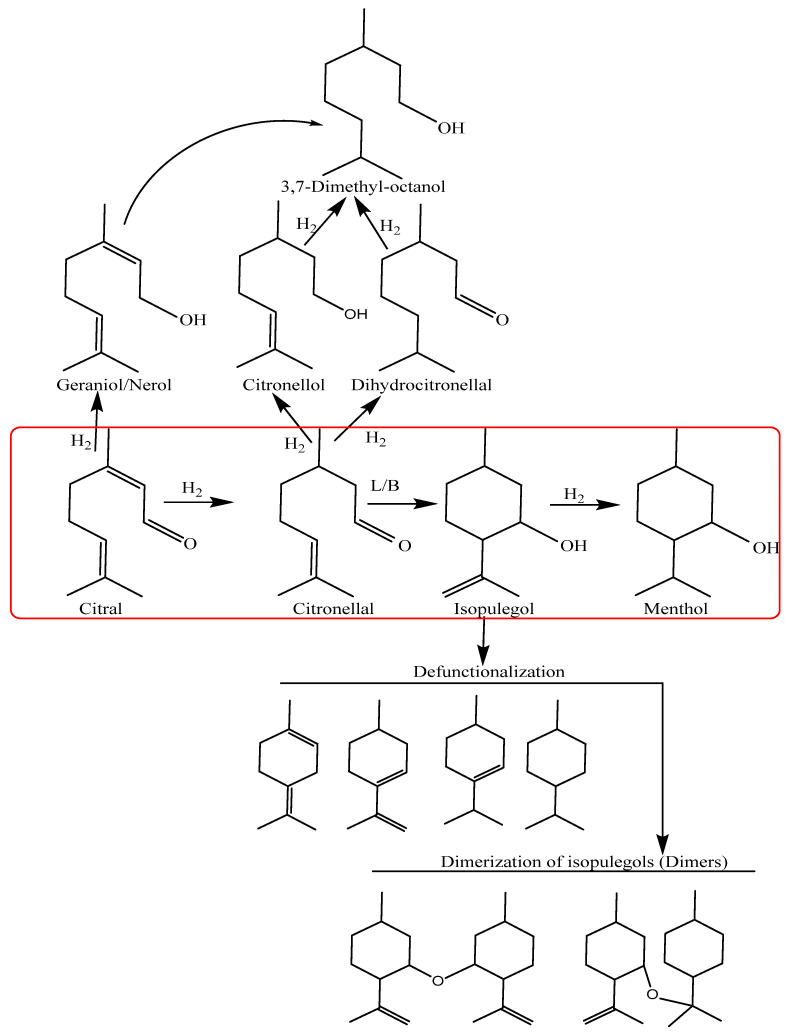
Reaction network of the transformation of citral to menthol and possible cracking reactions.

In the synthesis and catalysis field era, complex organic reactions are considered the most challenging tasks for sustainable, advanced, industrial (e.g., polymers, fine chemicals, fertilizers, petrochemicals, cosmetics, and pharmaceuticals) activities. Simultaneously, the catalytic hydrogenation of aldehyde compounds has been observed as the more interesting and challenging task for the production of fine chemicals [1]. R. Nayyori et al. [2] discovered a new reaction path for menthol synthesis using a 2,2′-bis(dipheny1phosphino)-1,1′-binaphthyl (BINAP) ligand catalyst. This catalyst helps in the isomerization of diethyl-geranyl-amine to citronellal, then hydrogenates to menthol. This complex reaction was considered the important reaction for menthol production at an industrial scale [3]. Among them, citral—or 3,7-dimethyl-2,6-octadienal—is known as an utmost versatile compound for the production of nerol, geraniol, isopulegol, menthol, citronellal, and dihydrocitronellal via consecutive hydrogenation and cyclisation catalytic reactions [4]. Likewise, in a catalytic citral hydrogenation reaction, the most important task is to produce menthol via one-pot synthesis from liquid-phase citral hydrogenation with the help of consecutive catalytic reactions, such as (i) citral hydrogenation to citronellal, (ii) citronellal cyclisation to isopulegol, and (iii) isopulegol hydrogenation to menthol [5,6], as shown in Scheme 1. The increasing world demand and price volatility of natural menthol have led to research on the possible synthesis of menthol from other raw materials. In 1998, Clark estimated the annual worldwide menthol production to be 11,800 metric tons, of which around 80% was natural menthol and 20% synthetic menthol. Currently, worldwide production is around 34,000 metric tons per year, with the share of synthetic menthol now being about 60%. This gives a clear indication of the growing importance of synthetic menthol [7,8]. Generally, it has been observed that a high yield of menthol production via citral hydrogenation would possibly be produced with the use of selective, active, and recyclable bifunctional (metal–acid) catalysts with selective properties. Various catalytic-material-supported (metals), such as H-MCM-41, Al-MCM-41, H-Y zeolite, beta zeolite, activated carbon, Zr-zeolites, ionic liquid catalysts, nickel-supported metals [9], MOF-derived AgCo alloy nanoparticles [10], Pt/Sn [11], Ru-HY extrudates [12], Pt/SiO_2_ [13] nano-composite catalysts [14], ruthenium nanoparticles [15], and SiO_2_-Al_2_O_3_, have been applied in citral hydrogenation reactions to obtain a higher yield of menthol [16,17]. Conversely, it has been observed that menthol selectivity would possibly be decreased due to the lack of active and selective catalyst designs that occur because of the formation of undesired/unrestrained hydrogenations, as well as cracking side reactions [5,6].

Among the innumerable superior catalytic materials, zeolite-based materials (e.g., Na-ZSM-5 and Na-Y) have been widely used for chemical organic reactions (e.g., hydrogenation, cracking, dehydration, and isomerization) [18]. However, previous literature reviews have highlighted the limitations of zeolite-based materials for bulky molecules/chemical reactants due to the narrow pore size of zeolite materials [5]. Additionally, the narrow pore size textural characteristics would also obstruct mass transfer, as well as the diffusion of reactant molecules, which would lead to the declination of catalytic reaction performance and product selectivity. In this regard, zeolite-based materials have displayed inadequate applicability in bulk organic reactions. Therefore, previous literature reviews have suggested various advanced post-synthetic methodologies (e.g., desilication reassembly, dealumination, templating, and mixed recrystallization) for the modification of the zeolitic framework/structure (e.g., wide mesopores and stable structures) in order to convalesce the rate of mass transfer, as well as the catalytic activity of the prepared materials in bulk organic reactions [18,19,20]. Additionally, these methods have been found more effective in the post-synthesis of micro-mesoporous zeolites (e.g., hierarchical zeolites) with maintaining a stable XRD framework structure [21]. Usually, hierarchical zeolites are more useful because they can be easily prepared by the treatment of commercial zeolites (e.g., Na-ZSM-5 and Na-Y) with alkalis and surfactants (this will be denoted by the “desilication reassembly process”) [21]. Chapelliere et al. (2019) [22] prepared hierarchical zeolites and studied the relationship between porosity, acidity, and catalytic properties in the catalytic cracking of bio-oil with a vacuum gas oil. They also developed a scale-up method [23] of alkali dissolution and a reassembly of ZSM-5 while changing the process variables such as the OH/ZSM ratio, the dissolution time, etc. However, there are still many uncertainties in the preparation of hierarchical catalysts for specific reactions. It has been suggested from previous experimental results and observations that the preparation of hierarchical zeolites using the desilication reassembly process with the addition of surfactants would lead to the growth of mesopores in the zeolitic microporous framework and strengthen the active acid site accessibility, which may help in catalytic performance enhancement in liquid-phase citral hydrogenation for one-pot menthol synthesis. However, applications of Ni-supported hierarchical zeolite bifunctional catalysts (Ni-HZSM-5 and H-USY) in citral hydrogenation reactions have not been applied yet to obtain a higher yield of menthols; therefore, we investigated the catalytic applications of metal-supported hierarchical zeolites for the first time in citral hydrogenation for the synthesis of menthols.

In this work, we attempted the preparation of metal-supported hierarchical zeolites (e.g., metal–acid bifunctional catalysts) and studied the effects of their preparation conditions on the catalytic performance in citral hydrogenation for menthol production. Herein, commercial zeolites (Na-ZSM-5 and Na-USY) were treated by varying the concentrations of alkalis (0.2–0.7 M NaOH) and surfactants (0.05 M CTAB) at the desired desilication temperature (80 °C). The physicochemical properties of nickel-supported hierarchical zeolites (post-synthesized zeolites) were investigated by instrumental analysis (e.g., X-ray diffraction, Nitrogen sorption, ICP-OES, H_2_-TPR, NH_3_-TPD, FE-STEM, hydrogen chemisorption metal dispersion, pyridine adsorption, and GC/GC-MSD techniques), and their relationship to the catalytic activities were examined in the hydrogenation reaction experiments. The catalysts thus prepared showed better performance in citral hydrogenation with a higher menthol yield, where the contributions of mass transport and those of acid and metal sites were analyzed.

## 2. Results and Discussions

The desilication reassembly process was used to investigate the structural changes and acidic characteristics of zeolites. ZSM-5, as well as USY zeolites, were desilicated with alkali concentrations at optimized conditions. During this alkali treatment, major changes were identified in porosity and structure, as well as acidity. To evaluate the catalyst properties, a number of characterization techniques, such as PXRD, FTIR, pyridine adsorption, NH_3_-TPD, and H_2_-TPR, as well as BET sorption, were carried out. We will study and review each characterization in detail to correlate catalyst characterization changes with catalytic activity and menthol synthesis. The detailed characteristics of Ni-supported hierarchical zeolite samples were determined and studied in detail.

### 2.1. Catalysts Characterization

**Figure 1:**
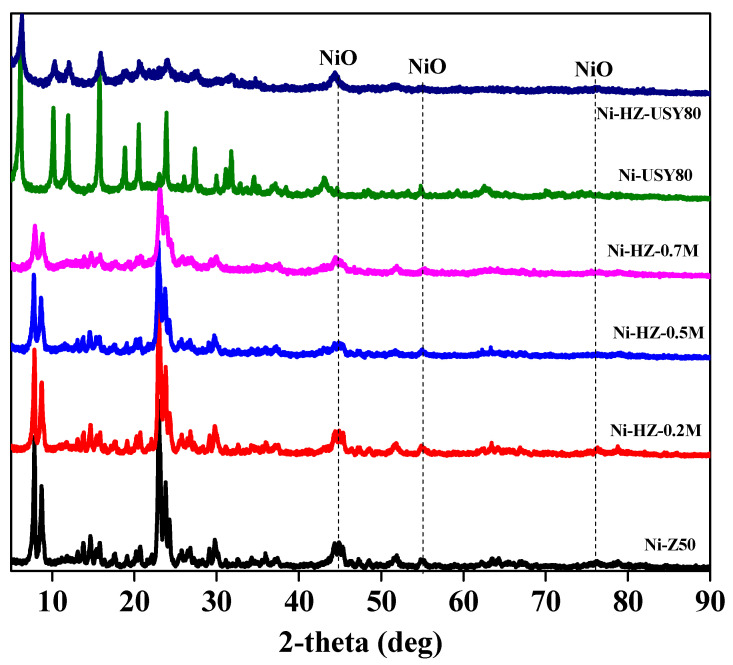
XRD patterns of Ni-supported hierarchical zeolites Ni-Z50; Ni-HZ-0.2 M; Ni-HZ-0.5 M; Ni-HZ-0.7 M; Ni-USY80, and Ni-HZ-USY80.

**Figure 2:**
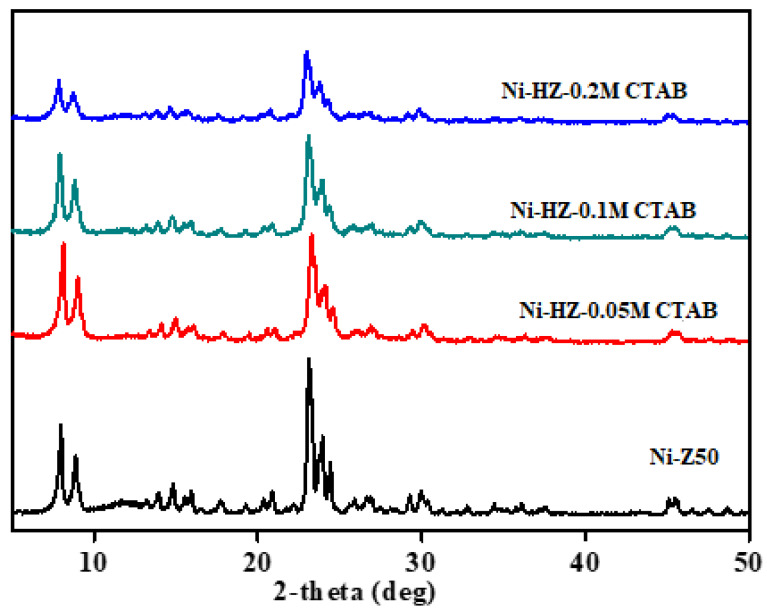
XRD patterns of nickel-supported desilicated zeolite samples Ni- Z50, Ni-HZ-0.05 M CTAB, Ni-HZ-0.1 M CTAB, and Ni-HZ-0.2 M CTAB. The desilication of ZSM-5 was carried out using a fixed amount of NaOH (0.5 M) at different concentration amounts of surfactant (e.g., hexadecyltrimethylammonium bromide). Whereas, 0.05 M, 0.1 M and 0.2 M showed CTAB surfactant concentrations which was used in desilication reassembly process.

XRD analysis of all prepared catalysts (nickel-supported) was conducted. The high crystal peaks were seen in the Ni/Z50 catalyst. The XRD peak intensity was drastically decreased with an increase in alkali concentrations. This change may indicate the loss of Si ions from the zeolite framework and weaken the zeolite XRD structure. The Ni-HZ-0.5 M catalyst was found as the most stable material after desilication treatment. NiO metal deposition was confirmed from XRD peaks at 44, 55, and 77°. Similarly, the USY80 zeolite was treated with alkali treatment (0.5 M NaOH and 0.05 M CTAB) and then doped with the nickel precursor. Ni-USY80 was found as the most stable material and possessed high-intensity XRD peaks, which were further decreased after alkali treatment. The reduction in XRD peaks confirms the loss of Si atoms and partial structural damage (Figure 1). Similarly, ZSM-5 zeolites were further desilicated at a fixed amount of NaOH (0.5 M) using different concentrations of the CTAB surfactant (0.05 M, 0.1 M, and 0.2 M). The effects of CTAB concentrations on the zeolite XRD structure are shown in Figure 2. A higher concentration of CTAB resulted in more dissolution of the zeolite framework and a destroyed crystal structure. The optimum CTAB concentration was 0.05 M. The effects of the CTAB surfactant were observed in the XRD structure, structure stability, and porosity enhancement. The effects of alkali treatment with respect to NaOH and CTAB concentration variations on zeolitic XRD structures were analyzed, as shown in Figure 1 and Figure 2. Higher concentrations of NaOH and CTAB have completely dissolved zeolite framework and caused more Si leaching. This effect has destroyed XRD crystal structure of zeolites. During optimization study, 0.5 M NaOH and 0.05 M CTAB concentrations were found optimum for obtaining hierarchical zeolite with a well-maintained XRD structure.

Furthermore, ICP-OES analysis of all nickel-supported catalysts was carried out. The amounts of Ni and Si/Al were analyzed in all zeolite samples. The amount of Si/Al is decreased during desilication reassembly process as shown in Table 1. Alkali treatment was conducted at different sodium hydroxide concentrations. The reduction in Si/Al means Si ions leached during desilication. The Si reduction amount confirms the desilication process, which was carried out successfully. Nickel loading was improved over the desilicated samples. ICP-OES analysis provided confirmation that nickel precursor particles were almost completely attached to zeolite surfaces.

Furthermore, BET sorption–desorption analysis was conducted for all nickel-supported catalysts, as shown in Table 1. The parent zeolite possessed a high amount of micropore volume and low mesopore volume. The values of the mesopore volume and surface area were drastically enhanced with alkali treatment. More severe Si leaching from the zeolite framework caused an increase in the mesopore volume and mesopore surface area. The original value of the micropore volume was continuously decreased with alkali treatment (Table 1).

**Figure 3:**
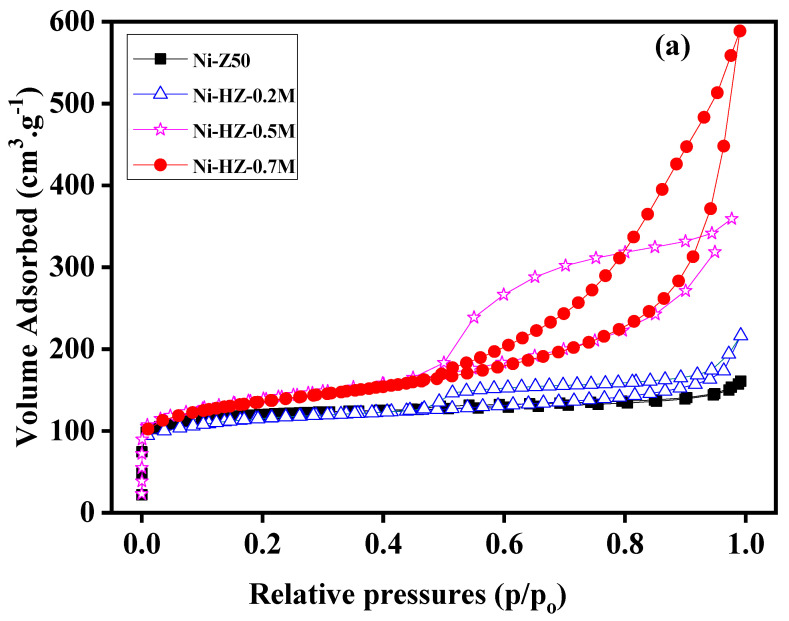

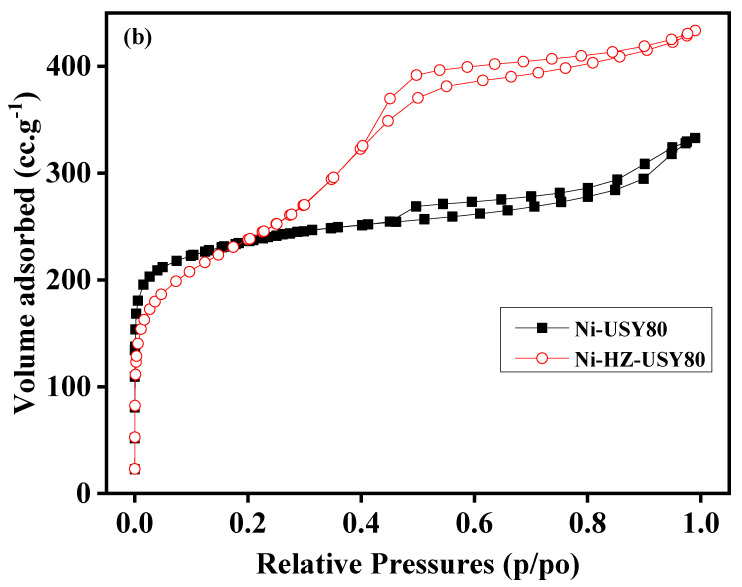
(**a**). shows nitrogen sorption isotherms of Ni-supported hierarchical ZMS-5 zeolite samples Ni-Z50, Ni-HZ-0.2 M, Ni-HZ-0.5 M, and Ni-HZ-0.7 M; whereas (**b**) shows nitrogen sorption isotherms of nickel supported hierarchical USY type zeolites samples (Ni-USY80 and Ni-HZ-USY80).

Similarly, BET sorption isotherm data of all samples (ZSM-5 and USY hierarchical zeolites) were revealed, as shown in Figure 3a,b. The sorption isotherm of Ni-Z50 (ZSM-5) may indicate the presence of micropores in the zeolite structure. With alkali treatment, the zeolite framework structure was reshaped, and its micropore characteristics were decreased, and new mesopores were formed inside the zeolite structure. This desilication process produces a number of wide mesopores in the zeolite framework, which helps in surface area enhancement (Table 1 and Figure 3a,b). According to IUPAC classifications, the two isotherms are of the IV shape with a H4 hysteresis loop, which is generally attributed to a hierarchical porous material containing both micropores and mesopores. The strong uptake at low relative pressure certifies the presence of micropores.

Furthermore, it was observed from BET and isotherm data that no more changes in textural properties were found in hierarchical zeolites after nickel doping. The ratio of Si/Al decreased with alkali concentration, which may enhance the mesopore surface area and mesopore volume, as shown in Table 1. Furthermore, it was observed that the Ni particle size was drastically increased with respect to alkali treatment. This size enhancement might be linked to wide mesopore formations.

**Figure 4:**
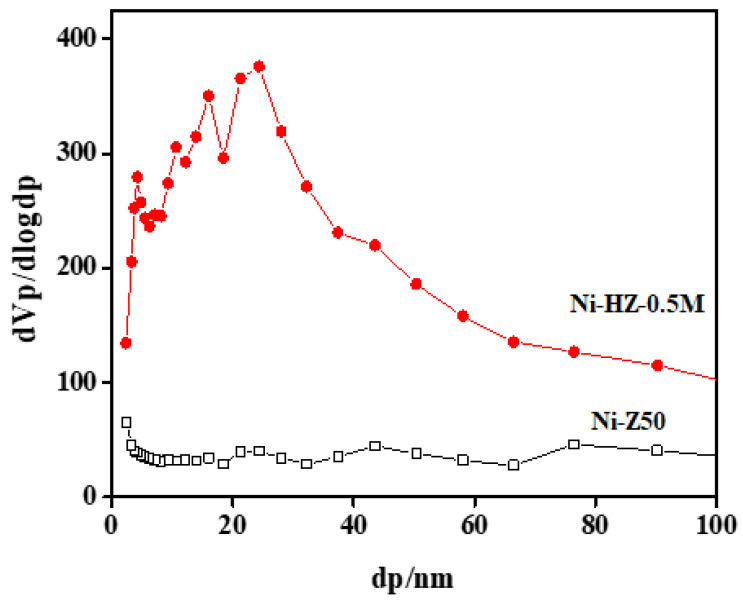
Pore size distribution (BJH) of parent and hierarchical zeolite samples.

BJH pore size distribution analyses of parent zeolite and hierarchical zeolite samples are given in Figure 4. The BJH pore size distribution curves of Ni-Z50 and Ni-HZ-0.5 M are shown in Figure 4. A major difference was observed in both samples. Parent zeolites possessed a purely microporous structure (e.g., a peak appeared at 2 nm), whereas new pore structure peaks appeared between the 5 and 35 nm range, which may confirm the formation of mesopores on the surface of zeolite frameworks after the desilication process, as shown in Figure 4. There was also a wide distribution of larger pores, stretching into the mesopore range up to 35 nm.

**Figure 5:**
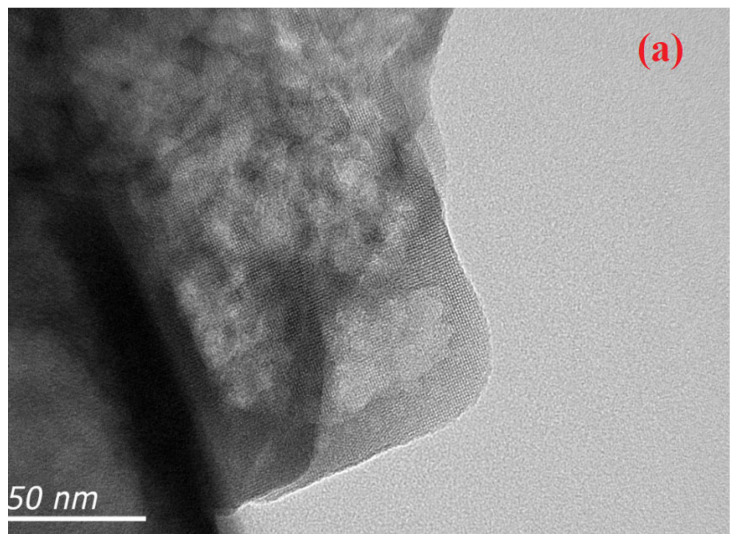

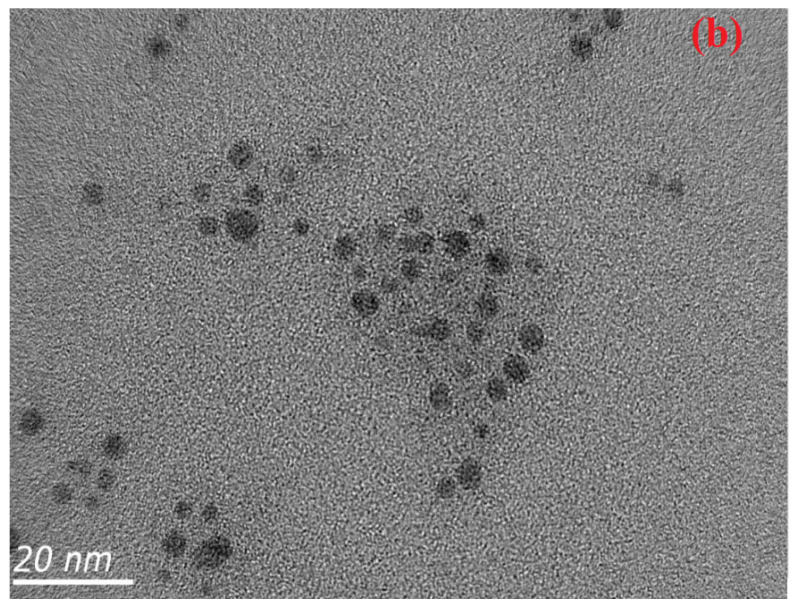
TEM images of (**a**) HZ-0.5 M and (**b**) Ni-HZ-0.5 M samples.

TEM analysis revealed the typical platelet-like morphology of the hierarchical zeolite (Figure 5). The TEM images show some morphological changes in the zeolite surface morphology. TEM analysis reflects the BET data that confirm mesopore formation. The black spots on the hierarchical zeolite surface may indicate the deposition of nickel particles, as shown in Figure 5b.

**Figure 6:**
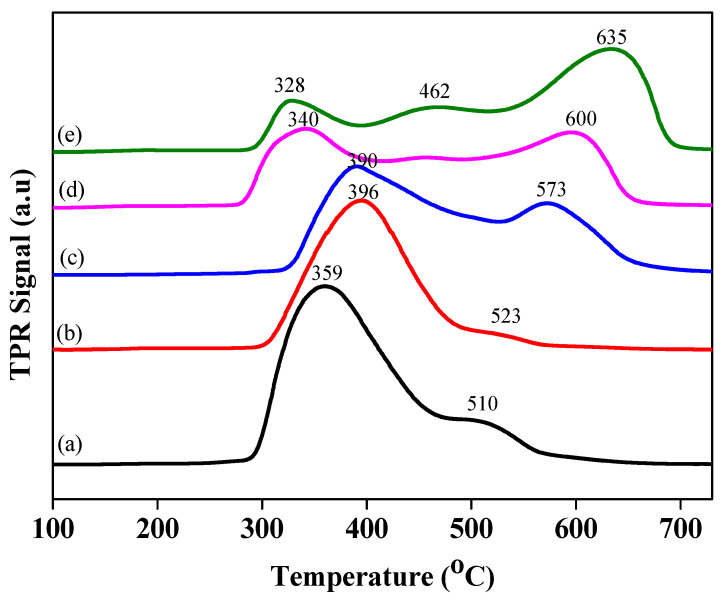
H_2_-TPR patterns of (a) Ni-Z50, (b) Ni-HZ-0.2 M, (c) Ni-HZ-0.5 M, (d) Ni-USY80, and (e) Ni-HZ-USY80 samples.

Further, H_2_-TPR analysis of all catalysts was conducted to evaluate the thermal changes in catalysts at different temperature ranges. TPR analysis, as well as nickel dispersion and particle size analysis of all samples, are provided in Figure 6 and Table 2. Low-temperature (LT) and high-temperature (HT) peaks can be seen in the TPR graph. This analysis reveals the presence of Ni^2+^ and NiO ions, as well as their interaction behaviors with hierarchical supports with reduction profiles at LT and HT peaks. The LT and HT peaks changed with alkali treatment. Temperature increase profiles were observed, as shown in Figure 6. The metal dispersion of Ni-supported hierarchical zeolite catalysts was assessed using the H_2_ chemisorption method, and the results are presented in Table 2. Ni particles were evenly distributed within the catalysts’ mesopores. The amount of Ni was dispersed over hierarchical zeolite surfaces because of the wider mesopores and high surface area (180–301 m^2^·g^−1^). The nickel metal dispersion rate was increased with the increase in mesoporosity and surface area. Nickel dispersion also had a connection with alkali treatment. It might be presumed that alkali treatment induced changes in the zeolite framework crystal structure and acidic properties.

Nickel dispersion over the parent and desilicated zeolites might have a negligible impact on surface properties. Nickel dispersion may slightly change metal and acid site strengths; its direct effects on catalytic activity and menthol selectivity were not observed. Similarly, nickel particle size variations may not have a direct impact on catalytic activity

**Figure 7:**
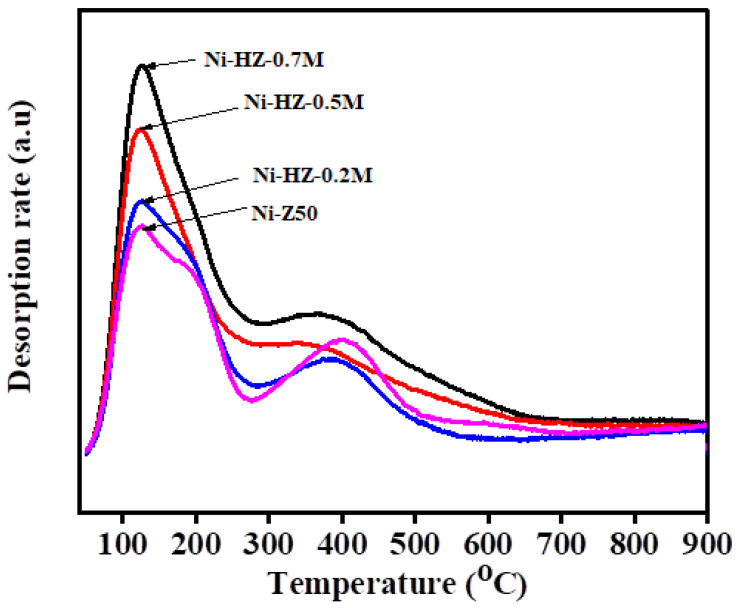
Ammonia TPD analysis of nickel-supported hierarchical zeolite samples.

**Figure 8:**
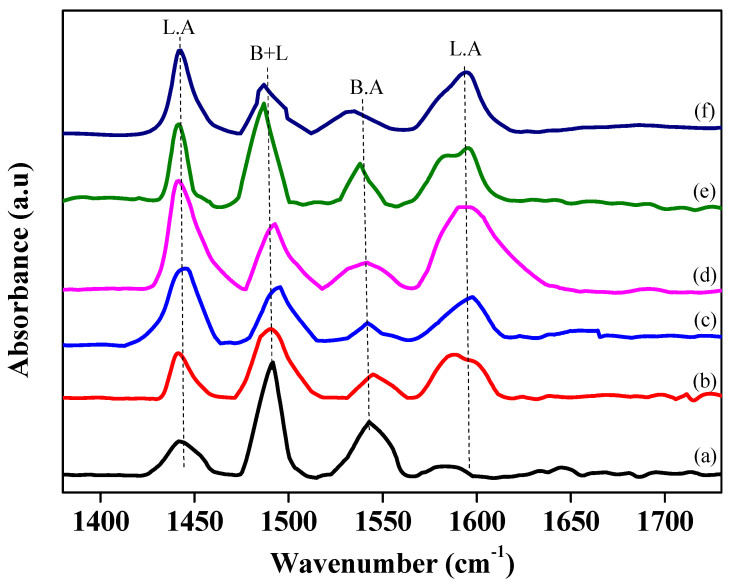
Pyridine-adsorbed IR spectra of hierarchical zeolite samples (a) Ni-Z50, (b) Ni- HZ-0.2 M, (c) Ni-HZ-0.5 M, (d) Ni-HZ-0.7 M, (e) Ni-USY80, and (f) Ni-HZ-USY80. Herein, LA and BA are known as Lewis acid sites and Bronsted acid sites.

Table 3 and Figure 7 and Figure 8 show pyridine adsorption FTIR and NH_3_-TPD characterization data of Ni-supported hierarchical zeolite catalysts. The acid strength and the number of acid sites are basic properties of solid acid catalysts/zeolites. The strength is probably measured by ammonia TPD, whereas the acid amount can be determined through the pyridine adsorption technique. The measurement of temperature-programmed desorption (TPD) and the heat of adsorption using a typical base, such as ammonia and pyridine, are the most useful techniques for evaluating the relative acid strength and the number of acid sites. Pyridine adsorption analysis also provided the quantitative and qualitative data of all zeolite samples. In the case of solid acid catalysts/zeolites, it is expected that the number of adsorption molecules will be equal to that of acid sites on the surface. It measures the nature and strength of acid sites. Additionally, we used NH_3_-TPD and pyridine adsorption techniques to evaluate the acidity characteristics of the prepared catalysts. The NH_3_-TPD profile showed high-temperature peaks as well as lower-temperature peaks. High-temperature peaks indicate strong acidic sites, whereas lower-temperature peaks indicate weak acid sites [24,25]. NH_3_-TPD and pyridine adsorption data show exceptional changes in acidity and acid site strength (Table 2 and Figure 7 and Figure 8).

Table 3 shows NH_3_-TPD and ICP-OES analysis data of catalyst samples. The amount of acidity (at low-temperature adsorption) improved with an increase in Al content. This Al content may be correlated with Si ion removal during the desilication process. Two curve peaks were recorded in the NH_3_ TPD spectrum: (a) from the total NH_3_ desorption at temperatures higher than 150 °C and (b) from the NH_3_ desorbed at temperatures higher than 390 °C, as shown in Figure 8. According to Hegde et al. [26], this distinction is necessary since, at lower temperatures, part of ammonia desorbs not from Bronsted sites but from structural defects, which are largely present in the hierarchical zeolites. It was, hence, assumed that the ammonia excess, in relation to the aluminum content, desorbed at temperatures between 150 °C and 390 °C would be adsorbed on structural defects. According to Jansen et al. [27], these defects are present in hierarchical zeolites due to local silicon unsaturated bonding, thereby introducing potential Lewis acid sites. Since stronger acid sites desorb ammonia at higher temperatures [28].

Characterization data suggest the presence of strong Bronsted and weak Lewis acid sites on the surface of the parent zeolite sample (Ni-Z50) [24]. After the desilication reassembly process, the strength of Lewis acid sites of zeolite was increased with Si leaching. The alkali treatment resulted in the creation of new Lewis acid sites, whereas the strength of Bronsted acid sites decreased, as shown in Figure 8 and Table 3. This drastic change in acid sites might be due to Si leaching from the zeolite framework and an increase in Al content. This increase in Al content may cause more increases in total acidity, as shown in NH_3_-TPD data (Table 3). This acidity enhancement is correlated with the nature of acid sites and their strength. After the desilication process, the Ni-HZ-0.5 M catalyst exhibited strong Lewis acid sites and medium Bronsted sites as compared to the Ni-Z50 catalyst, whereas the Ni-HZ-0.7 M catalyst exhibited strong Lewis acid sites as well as weaker Bronsted acid sites.

The sudden enhancement in Lewis acid sites is probably interconnected with Si leaching and Al content deposition on the zeolite exterior surface. NH_3_-TPD and pyridine adsorption analysis data reveal that total acidity and acid site ratios were increased with alkali treatment. Similarly, the pyridine adsorption characterization of the USY zeolite and its hierarchical zeolite catalysts were carried out to evaluate the nature and strength of the acid sites. The parent USY zeolite contained strong Bronsted and weak Lewis acid sites on its surface. During desilication treatment, there were drastic changes in Lewis/Bronsted sites. The strength of Lewis acid sites improved after alkali treatment, as shown in Table 3. USY zeolite samples contained a high amount of acidity as compared to ZSM-5 samples. Whereas Bronsted sites drastically decreased during desilication treatment. The quantity of Lewis acid sites were improved as compared to Bronsted acid sites. It can be assumed that this type of radical change in acidity and acid sites may introduce some changes in the overall catalytic performance and menthol synthesis. This reaction might be connected to mesopores and acid sites. This reaction probably took place through physical surface adsorption into mesopores and interaction with active acid sites.

### 2.2. Synthesis of Menthol from Citral Hydrogenation

**Figure 9:**
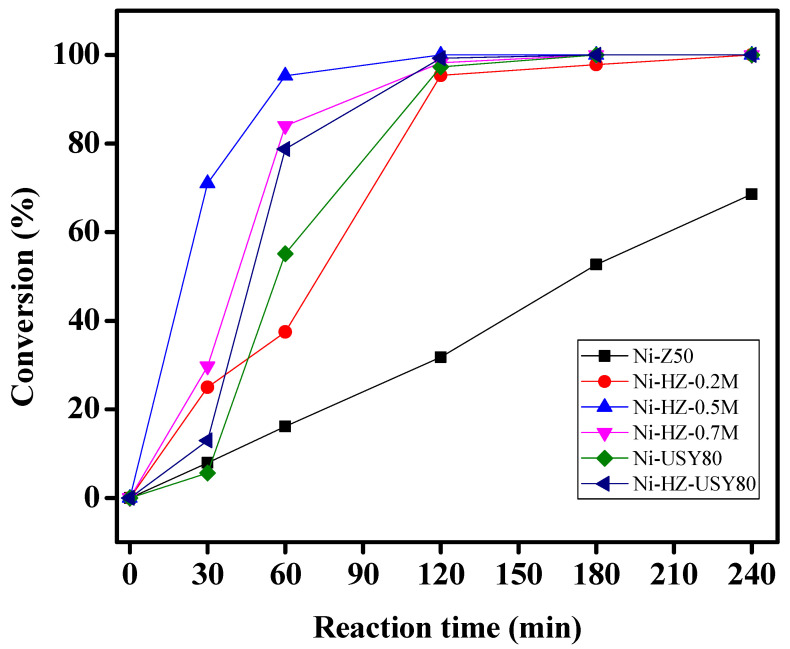
Catalytic performances of bifunctional Ni-supported hierarchical zeolite catalysts in citral hydrogenation reaction with reaction time.

Citral was hydrogenated in an autoclave batch reactor at 100 °C and 1.0 MPa hydrogen to investigate the characteristics of Ni-supported hierarchical zeolite catalysts. The reaction performance of all catalysts is reported in Table 4. Citral hydrogenation produced the primary compounds, including citronellal, isopulegol, menthol, citronellol, and 3,7-dimethyl octanol, with only a minor number of dehydrated products produced. Figure 9 highlight the catalytic activities of various catalysts during the hydrogenation of citral as well as changes in the catalytic activity product distribution and citral conversion over time. It is interesting to note that citronellal and isopulegol were produced at the first stage of hydrogenation before being converted to menthol or citronellol.

**Figure 10:**
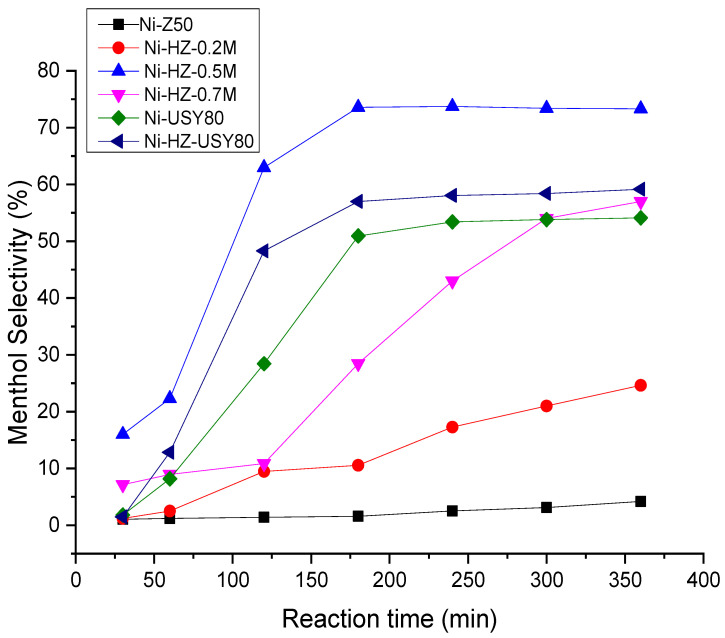
The catalytic performance of catalysts in citral hydrogenation for menthol synthesis with respect to time via citral hydrogenation.

Similarly, Figure 10 shows the catalytic performance of various catalysts for menthol production. All hierarchical zeolites showed different catalytic performance in menthol synthesis. The surface, as well as the acidic properties of all desilicated zeolites, drastically changed with respect to alkali treatment. The Ni-Z50 catalyst was totally microporous in nature; its mass transfer rate was the lowest when compared to the other samples. This catalyst probably could produce menthol less than 8% within 24 h. Specific textural changes were observed in the parent zeolite after alkali treatment; its surface and acidic properties were enhanced, which helped in catalytic activity enhancement and menthol formation, as shown in Figure 10. Among these catalysts, the Ni-HZ-0.5 M catalyst could produce 83% menthol within 300 min. By comparing the characterization and reaction data, catalytic activity and menthol formation have no direct link to Ni particle size.

**Figure 11:**
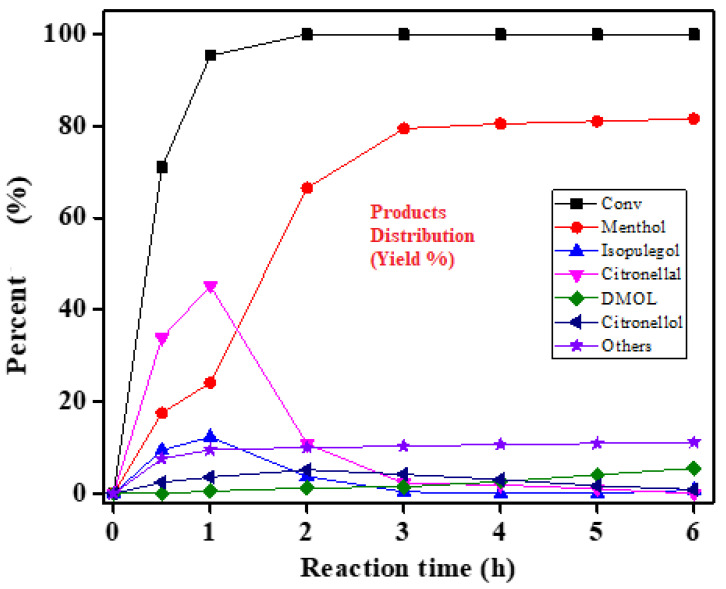
The conversion and product distribution of citral hydrogenation given by nickel-supported hierarchical zeolite catalyst (Ni/HZ-0.5 M).

Table 4 illustrates that the Ni-supported hierarchical zeolite (Ni/HZ-0.5 M) catalyst outperformed other catalysts in the citral hydrogenation reaction, achieving 100% conversion in 1 h and a TOF of 7.12 h^−1^. After 1 h, the rate of menthol synthesis increased quickly, yielding 83% of menthol after 3 h. Citronellal converted to isopulegol more quickly than other catalysts, although subsequent hydrogenation to menthol happened more slowly. Mass transfer rates, selectivity, and reaction rates all improved. Without favoring alternative hydrogenation processes, this catalyst enabled the direct conversion of citral to citronellal and, finally, isopulegol (by roughly 94%). (Figure 11). Due to the dehydration of isopulegol, some amounts of fractured products were produced with reaction time. The catalytic activity was specifically in the range Ni-HZ-0.7 M > Ni-HZ-0.5 M > Ni-HZ-0.2 M > Ni-Z50.

The desilication reassembly process of zeolite may bring structural changes that may affect catalytic activity and selectivity. XRD and BET analyses reveal the formation of micro-mesoporous structures. The narrow pore structures cause the poor mass transfer of molecules, the accumulation of molecules (lower diffusion), and less accessibility to acid sites as well as active sites of catalysts. Purely micro porous nature zeolite, ZMS-5 (Ni-supported Z50) exhibited poor catalytic performance and produced lower amount of menthol in one pot reaction, this poor performance might be connected with zeolite narrow pores that resulted low diffusion. Ni-Z50 has presumed poor catalytic activity (TOF 0.8 h^−1^), and menthol formation was 4%. However, the desilication reassembly process might have induced porosity structure changes that have a close relation to catalytic activity and menthol production enhancement. The hierarchical zeolite-supported nickel had enhanced catalytic activity, which was increased from 0.8 TOF h^−1^ to 4.75 TOF h^−1^. This resulted in full substrate conversion and produced 83% menthol within short time (3 h). The excellent catalytic performance and fast diffusion was achieved in hierarchical zeolite samples; this change might be connected with high mesoporosity and balanced metal-acid sites (Figure 9, Figure 10 and Figure 11 and Table 4). Catalytic performance and selectivity are majorly connected. This hierarchical zeolite introduced an excellent change in structural and porosity properties, which improved catalytic performance, reaction rate, and product selectivity characteristics.

It was revealed that the desilication reassembly process of zeolite induces novel changes in the microporous structure and acidic properties. The reactant molecules may not have access to active acidic sites for the reactant because of the narrow micropore structure. The molecules’ approach is negligible, which causes lower catalytic activity and product formation. It was revealed from previous studies that the presence of acid sites over the catalyst surface is necessary for the transformation of citronellal to isopulegol, then menthol. The easy access of reactant molecules to active acidic sites enhanced catalytic activity and menthol product formation. The easy molecular access to acid sites is probably because of wide pore formation and an increase in surface area. These structural and acidic changes might have some effects on the reaction performance of one-pot menthol synthesis. It was revealed that the total acidity strength of the catalyst may be closely interconnected to mesoporosity enhancement. Similarly, the enhancement in acidity improved catalytic performance in liquid-phase citral hydrogenation. Textural and reaction performance characteristic data are shown in Table 1, Table 2, Table 3 and Table 4. Wider mesopores probably provide more accessibility of reactant molecules to active inner and outer acidic sites of the catalyst. It was exposed that these acid sites may develop during aluminum ion deposition on the exterior surface of the zeolite framework, which create new Lewis acid sites (e.g., on Si leaching from the zeolite framework by use of the desilication reassembly process). Experimental data justified the improved catalytic performance (TOF from 0.8 to 7.14 h^−1^) and menthol selectivity (~83%), which are directly interlinked with enhanced acidity and mesoporosity characteristics (Table 1, Table 2, Table 3 and Table 4).

The types and nature of acidic sites (e.g., Lewis and Bronsted) have a great influence on catalytic activity and selectivity enhancement. The strength of acid sites is considered a major problem in catalyst design based on the reaction mechanism. A zeolite is a narrow micropore structure and possesses strong Bronsted acidity. The Si leaching process (desilication reassembly process) changed the acidity strength and L/B ratio. The strength of Lewis acid sites improved during Al deposition on the zeolite extra framework. Similarly, the strength of Bronsted acidity decreased, whereas Lewis acidity strength increased. During the preparation of the hierarchical zeolite, Lewis acidity was stronger than Bronsted acidity, which may promote citronellal cyclisation to isopulegol, then hydrogenation to menthols. An experimental study and previous study predicted that strong Bronsted acidity may cause the cracking or dehydration of isopulegol/menthol-based cyclic compounds, which may result in a reduction in menthol production. However, Lewis acid sites of the hierarchical zeolite were enhanced as compared to Bronsted sites (e.g., total acidity increased from 1.25 to 2.12 mmol·g^−1^) (Figure 8 and Table 4). The poor performance of the parent zeolite in citronellal cyclisation might be due to the presence of micropores and Bronsted acid sites. Although, the new Lewis acid sites formed due to the creation of wider mesopores inside the zeolite framework.

Experimental data (Table 4 and Figure 9 and Figure 10) indicate that Ni-supported zeolites (Ni/50 and Ni/HZ-0.2 M) possess poor catalytic performance in citral hydrogenation for one-pot menthol synthesis; this might be due to the presence of micropores, less surface area, and weak Lewis acid sites. The menthol production rate improved with an increase in Lewis acidity after alkali treatment. Higher concentration of Lewis acid sites on the hierarchical zeolite surface has been achieved due to Si leaching, However, the Ni/HZ-0.5 M catalyst produced 83% menthol at full citral substrate conversion within a short time (~3 h) in the presence of strong Lewis and weaker Bronsted acid sites (Table 3 and Table 4). The formation of wider mesopores results in an increase in Lewis acid sites and provides easy accessibility to substrate molecules for fast and desired reactions [29].

Similarly, the effects of nickel metal dispersion on the surface of hierarchical zeolites were studied, and their correlation with catalytic activity and menthol selectivity was determined. It was observed from experimental and characterization data that the nickel dispersion rate improved with the formation of mesopores and high surface area. The metal dispersion has a close interaction with the mesoporosity and acidity strength characteristics that may affect (improve) catalytic activity and selectivity. The metal dispersion with mesoporosity and acidity properties showed different catalytic behavior in one-pot menthol synthesis. When the nickel metal was doped over the parent zeolite (Ni/Z50), it showed less metal dispersion on the surface of the zeolite, which resulted in poor catalytic performance in the one-pot menthol synthesis reaction. It may be assumed that this weak catalyst performance might be due to the unavailability of mesopores and weak acidity, which restricts facilitating mass transfer or fast diffusion. In this era, an additional characterization study was carried to find out the effects of nickel metal dispersion on the hierarchical zeolite surface. The nickel dispersion rate was drastically enhanced in the hierarchical zeolite (4.99%) as compared to the parent zeolite (2.95%). This drastic improvement might be related to wide mesopores and high surface area (180~301 m^2^·g^−1^) and the decrease in crystallite size (34.28~20.25 nm), as shown in Table 1 and Table 2. On the utilization of the nickel-dispersed hierarchical zeolite (Ni-HZ-0.5 M) in liquid-phase citral hydrogenation, it gave outstanding catalytic performance and menthol synthesis. Metal particles were widely distributed in the mesopores of the hierarchical zeolites, which may facilitate hydrogenation and cyclisation reactions with fast mass transfer/diffusion. The presence of Ni active particles facilitates citral hydrogenation to citronellal, whereas balanced acid sites (L/B) help citronellal cyclisation to isopulegol. Furthermore, isopulegol molecules were hydrogenated to menthols over Ni metal sites. When specific amounts of nickel metal (8 wt %) were doped over various hierarchical zeolites (HZ-0.2 M, HZ-0.5 M, and HZ-0.7 M), various distribution rates were observed, along with dissimilar reaction behaviors.

It was examined from pyridine adsorption data that the strength of Lewis and Bronsted acid sites varied after the desilication reassembly process of zeolite. The number of Lewis acid sites increased in the formation of the hierarchical zeolite. Furthermore, the high dispersion of Ni metal particles over the surface of the hierarchical zeolite may change the strength of acidic sites, and a balanced ratio of metal–acidic sites was achieved, as shown in Figure 8 and Table 3. It was revealed that Ni doping on the prepared hierarchical zeolites (e.g., treated with alkali treatment at different NaOH concentrations) gave a variable Ni dispersion percentage on the hierarchical zeolite. This variable Ni dispersion ratio changed the catalytic behavior and performance. The overall catalytic performance of all prepared Ni-supported catalysts is mentioned in Table 4. This drastic change is mostly related to the ratio of metal–acid sites. This change has a large impact on citral conversion and menthol yield. The optimum catalytic performance and menthol yield is seen as possible because of balanced metal–acid sites and mesopore formation. The product distribution of citral hydrogenation to menthol is shown in Figure 11. Ni-HZ-0.5 M hierarchical based catalyst exhibited an excellent catalytic performance and higher menthol yield. A high nickel dispersion rate was found in the Ni-HZ-0.7 M catalyst because of its high mesoporosity, but the XRD structure was partially damaged. The catalytic menthol production (47% menthol yield) was recorded with the use of the Ni-HZ-0.7 M catalyst, which was much lower than the Ni-HZ-0.5 M catalyst performance. From the experimental and characterization data, it was observed that balanced metal–acid sites were maintained, which promotes hydrogenation, as well as cyclisation reactions. This helps in improving menthol selectivity. The rates of cracking and dehydration side reactions are controlled because of balanced acid sites (L/B).

In addition, NH_3_-TPD analysis was carried out and estimated the total acidity of all prepared catalysts. The acidity of the Ni/Z50 catalyst was 1.25 mmol·g^−1^, which was further enhanced after desilication reassembly treatment (Table 3). However, more leaching of Si ions from the zeolite framework caused an increase in acidity. The acidity increase may have an interconnection with the Al content. The Ni metal dispersion increased according to the pore size and surface area. The enhancement in surface and porosity properties would have direct/indirect impact on the Ni dispersion rate. Similarly, a H_2_-TPR (temperature-programmed reduction) analytical characterization of all prepared catalysts was carried out to examine the surface chemistry of metal oxides under thermal conditions. The low-temperature (LT) and high-temperature (HT) parameters of Ni/Z50 are 359 and 510 °C, respectively. These temperature parameters are increased (e.g., increased with alkali concentration treatment) with an increase in mesoporosity and acidity. It can be examined from the available data that the hierarchical zeolite supported with highly dispersed nickel (HZ highly dispersed Ni catalyst (Ni/HZ-0.7 M)) gave medium menthol selectivity (47%), which assisted the hydrogenation of citronellal to citronellol, as can be seen in Table 4. It is assumed that active acid sites may be covered by metal sites due to zeolite narrow structure. Due to narrow pore structure, reactant molecules movement might be slow and all molecules may have accumulated into narrow pores and might have negligible access to active acid sites of zeolites that resulted low mass transfer rate and poor catalytic activity. There was no direct access for the reactant molecules to the active acidic sites of zeolites, which might be a due to the partial blockage of active acid sites by nickel ions. Therefore, the rate of cyclisation was found to be lower when using the nickel-supported parent zeolite as compared to the hierarchical zeolites. Moreover, when parent zeolites were transformed into hierarchical zeolites, this introduced wide mesopores and balanced metal–acid sites, along with an improved mass transfer rate. These types of textural changes brought improvements in mass transfer and reaction rates, catalytic activity, and selectivity. The balanced metal–acid site ratios were obtained with the desired dispersion of Ni particles over the surface of the hierarchical zeolites (Ni-HZ-0.5 M). This catalyst exhibited higher catalytic activity (TOF 7.12 h^−1^) and menthol production (~83%), which facilitated a cyclisation reaction of around 94% [30], which is more consistent with the product distribution of citral hydrogenation (Figure 11). The overall catalytic performance and menthol formation under optimized conditions are majorly connected with improved mesoporosity, acidity, balanced acid site ratios, and nickel dispersion characteristics.

**Figure 12:**
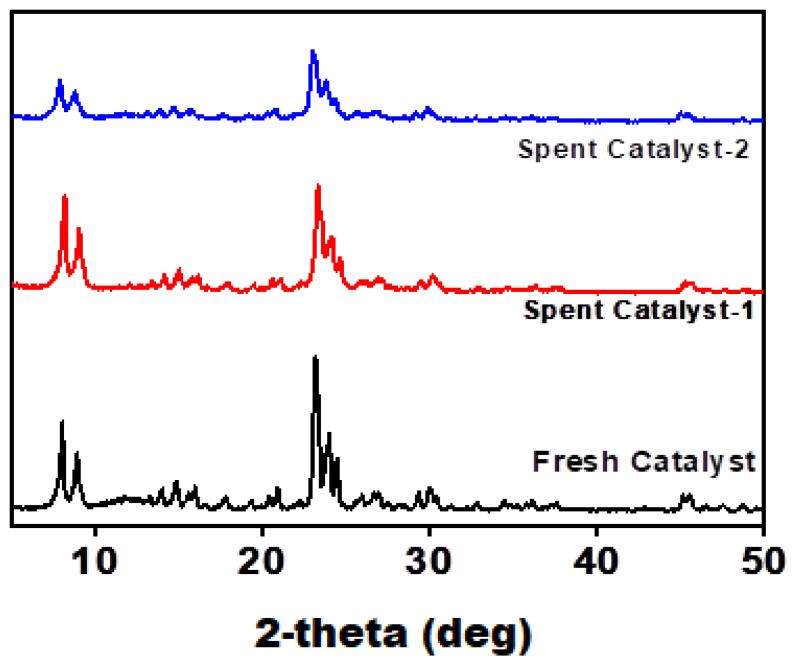
XRD patterns of Ni-HZ-0.5 M catalyst during recyclability test.

After a detailed experimental and characterization study, the recyclability test of the best catalyst (Ni-HZ-0.5 M) was evaluated. In the first reaction cycle, the catalyst gave 100% conversion and 83% menthol selectivity. After one reaction cycle was complete, the catalyst was filtered and washed with a toluene solvent and dried in an oven for 8 h and then reused in the second reaction cycle. However, the second reaction cycle gave an almost similar performance, as shown in Table 2. A similar procedure was conducted for catalyst regeneration and reused in the citral hydrogenation reaction. The reaction proceeded for 3 h. The third cycle also gave an almost similar reaction performance. During the recyclability test, the catalyst (Ni-HZ-0.5 M) was found to be the more stable and active catalyst for citral hydrogenation for menthol synthesis (Table 5). During the recyclability test of the best catalyst, XRD characterization was carried out and then compared with catalytic activity. It was observed that the catalyst remained stable in the three consecutive reaction cycles and gave an almost similar reaction performance. The XRD patterns remained almost similar in the consecutive recycle tests, as shown in Figure 12.

### 2.3. Comparative Study with Other Catalysts

**Figure 13:**
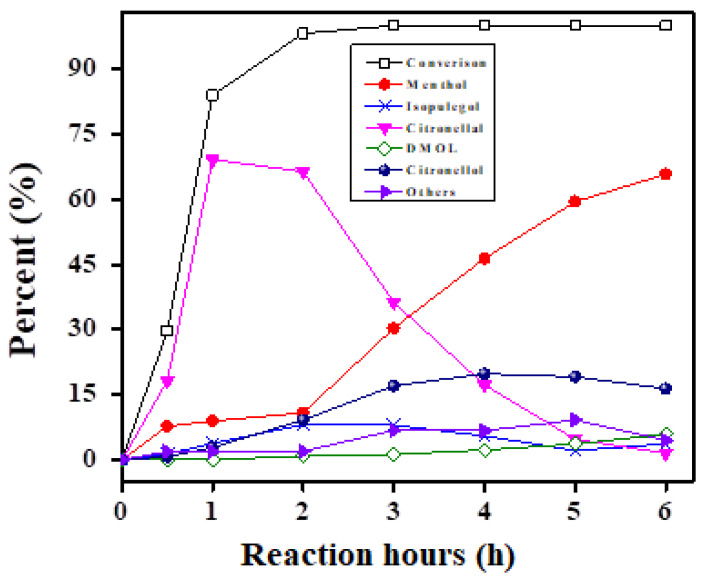
Product distribution of citral hydrogenation reaction over Ni-supported hierarchical USY catalyst (Ni-HZ-USY80).

For the evaluation of the catalytic performances of other zeolites with ZMS-5 and its hierarchical zeolites, a comparative study was carried out. In this study, USY (Si/Al 80) was chosen as the comparative zeolite catalyst. Similar optimum desilication process parameters were used in preparation of hierarchical zeolites. A similar characterization of USY and its prepared hierarchical zeolite was carried out after nickel impregnation. The characterization data showed an almost similar trend in physicochemical and catalytic properties as compared to Ni-HZ-0.5 M. In addition, the Ni-USY80 and Ni/HZ-USY80 catalysts exhibited good catalytic performances (e.g., the catalytic activity of Ni-USY of TOF 0.56 h^−1^ was enhanced to 1.30 h^−1^ after the desilication process); these drastic changes might be due to the extra increase in the mesopore surface area (260~730 m^2^·g^−1^) and total acidity (2.42~2.54 mmol·g^−1^), as can be seen in Table 1, Table 2, Table 3 and Table 4. In the case of USY and the hierarchical-based catalyst, metal dispersion had no direct connection with catalyst activity enhancement. The strength of Bronsted acidity decreased in the preparation of hierarchical zeolites. The metal dispersion did not show any effect on menthol selectivity enhancement when using the USY zeolite, whereas it was improved about 58% due to the decrease in the Bronsted acid sites of the hierarchical USY support (Figure 8), and the cracking rate decreased. It was observed that around 40% isopulegol was cracked into side products in the presence of stronger Bronsted acid sites (e.g., Ni-USY80), which results in lower menthol production (Figure 13). No more mass transfer limitation issues were rectified in the USY zeolite reaction study.

Table 6 shows a comparative study of various bifunctional metal–acid catalyst performances in menthol synthesis. The different nature of acidic supports, such as beta zeolites, alumina, ALPO, heteropoly-acid-supported alumina, MCM-41, and montmorillonite clay, were impregnated with metals and applied in citral hydrogenation. All zeolitic-type catalysts have shown good catalytic conversion but these catalysts were limited to menthol synthesis. Menthol selectivity was achieved in the range of 2 to 75%. In our research study, zeolite ZSM-5 was desilicated and transformed into a hierarchical (micro-mesoporous) zeolite at optimum alkali treatment conditions, which showed excellent catalytic activity and the highest menthol yield as compared to other reported catalysts, as shown in Table 6.

In summary, it can be precisely concluded that the desilication reassembly process was found as the most suitable option for the formation of hierarchical zeolites (micro-mesoporous zeolites) at optimum process conditions (0.5 M NaOH, 0.05 M CTAB, and 12 h reaction). The catalytic performance of purely microporous zeolites was further improved in citral hydrogenation after the desilication step. This process improved the mesoporosity, total acidity, and metal dispersion, which helped the enhancement in catalytic activity and menthol selectivity. After the desilication process and Ni doping, metal and acid sites were improved and balanced, which controlled the citronellal cyclisation and isopulegol hydrogenation reaction rates for the optimum menthol yield. Strong Lewis acid site and weaker Bronsted acid site ratios were found for perfect cyclisation. High surface area, high mesopore volume, high acidity, and strong Lewis acid sites were the main parameters for obtaining the highest menthol selectivity. Among these catalysts, a bifunctional metal–acid (Ni/HZ-0.5 M) catalyst was the more promising catalyst for the liquid-phase citral hydrogenation reaction for one-pot menthol synthesis to obtain a higher yield of menthol with an improved mass transfer rate.

## 3. Materials and Methods

### 3.1. Catalyst Preparation

#### Hierarchical Zeolite (Acidic Support) Preparation

**Scheme 2:**
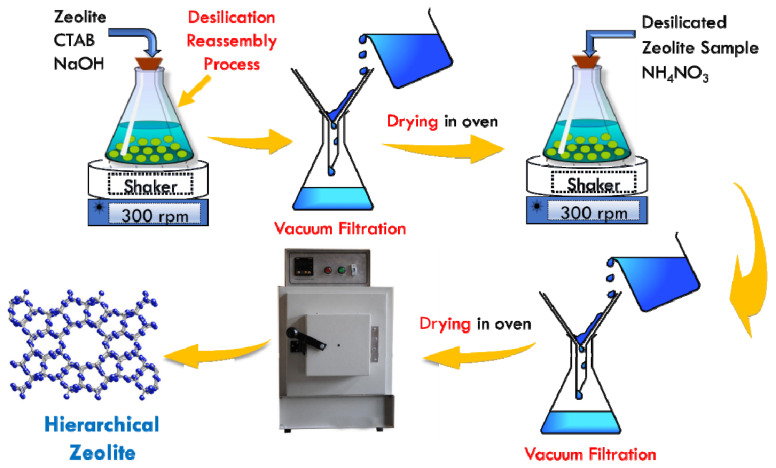
The diagram shows post-synthesis steps of the hierarchical zeolite catalyst using desilication reassembly process.

Commercial zeolites (e.g., ZSM-5 and USY) were purchased from Zeolyst International, USA. In order to understand how to prepare hierarchical ZSM-5 and USY, desilication reassembly of the ZSM-5 and USY zeolites was attempted with various combinations of NaOH concentrations, alkali treatment temperatures, and CTAB concentrations [21] at the conditions used in other previous studies. The experimental procedure is provided in graphical Scheme 2. The desilication of zeolites may bring major structural properties into zeolite frameworks on Si atom leaching. The ZSM-5 zeolite was desilicated at various optimization conditions, such as alkali concentrations (0.2, 0.5 M, and 0.7 M NaOH), CTAB surfactant concentrations (0.05 M, 0.1 M, and 0.2 M), and desilication temperatures (40 °C, 60 °C, and 80 °C). The number of steps were followed to optimize desilication reassembly treatment. This optimization was correlated with structural properties, catalytic performance, and stability.

For the catalyst’s preparation, aqueous mixtures of NaOH (0.2 M/0.5 M/0.7 M, 20 mL/g zeolite) and CTAB surfactant (0.05 M, 20 mL/g zeolite) were poured into a glass reactor (2000 mL), mixed (through an agitator), and heated (controlled heater) to achieve the specific temperature of the desilication process, respectively. On attainment of the optimum desilication temperature, the measured amount of microporous ZMS-5 zeolite in powder form (CBV-5524 G) was added immediately into the glass reactor, and the desilication reaction was initiated through stirring (at constant speed of 300 rpm) and stirring was continued for 12 h at a constant temperature (80 °C) under water reflux conditions. After 12 h, the desilication process was stopped using the water ice quenching process. Desilication reaction could be stopped below −5 °C temperature. After the quenching process, reaction slurry was filtered, washed with distilled water, and dried at 120 °C in the lab oven for 24 h. The desilicated zeolite samples were calcined in muffle furnace at 550 °C for 5 h under a heating rate of 5 °C/min in the presence of air for the deformation of the surfactant layer and mesopore formation. Afterwards, to obtain a proton form of hierarchical zeolite, the desilicated sample was further ion-exchanged with ammonium nitrate (0.5 M NH_4_NO_3_, 20 mL/g zeolite, 60 °C, and 2 h) three times under reflux conditions, then washed, dried, and calcined at 550 °C for 5 h. The zeolite was treated with NaOH using a concentration of 0.2 M, 0.5 M, and 0.7 M, respectively (at a constant 0.05 MCTAB concentration). These calcined samples were labeled as HZ-0.2 M, HZ-0.5 M, and HZ-0.7 M; Whereas, HZ coding represents to hierarchical zeolite, whereas 0.2 M, 0.5 M, and 0.7 M represent alkali concentrations.

In a similar way, the hierarchical zeolite USY (Si/Al = 80) was prepared using the desilication reassembly process according to above-mentioned procedure. This type of desilicated sample was named HZ-USY80.

### 3.2. Preparation of Nickel-Supported Hierarchical Zeolite Bifunctional Catalysts

Bifunctional metal–acid catalysts were prepared through the impregnation of nickel nitrate hexahydrate precursor (8 wt %) over hierarchical zeolite acidic supports (HZ-0.2 M, HZ-0.5 M, and HZ-0.7 M) using the wet impregnation method. Nickel nitrate hexahydrate precursor was dissolved into 20 mL of ethanol solvent, doped drop-wise on the surface of zeolitic slurry, and stirred at room temperature for 24 h at a constant speed (300 rpm). Wet impregnated samples were dried in a vacuum rotary evaporator at 60 °C for 12 h. Furthermore, impregnated catalysts were calcined in a muffle furnace at 550 °C for 4 h at a fixed temperature rise ramping program. Subsequently, Ni-supported catalysts were activated in a glass tubular furnace at a temperature of 500 °C under a constant pressure and flow of hydrogen gas (20 mL/min). This catalyst reduction was continued for 4 h using a temperature ramping program. At the end of the catalyst reduction step, catalysts were purged with argon gas (99.99%, pure) for 30 min. The freshly reduced catalysts were used in citral hydrogenation reactions. These activated catalysts were branded as (8 wt %) Ni-Z50, Ni-HZ-0.2 M, Ni-HZ-0.5 M, Ni-HZ-0.7 M, Ni-USY80, and Ni-HZ-USY80). In addition, Z50 and USY80 were parent zeolite samples, whereas after desilication reassembly process of these parent zeolites, hierarchical zeolites (e.g. HZ-0.2 M, HZ-0.5 M, HZ-0.7 M, and HZ-USY80) were prepared.

### 3.3. Catalyst Characterization

The physical properties of the prepared catalysts were determined using characterization techniques. However, the structural properties of parent zeolites and hierarchical zeolites were measured using an X-ray diffractometer (XRD-6000, Shimadzu, Kyoto, Japan) at different range of 2θ (5~90°). Operating parameters, such as app. current (100 mA) and app. voltage (40 kV), were fixed during the machine operation. Similarly, elemental composition of zeolite samples was analyzed with ICP-OES technique. Before ICP analysis, zeolite samples in powder form (quantity of 0.1 g) were dissolved/digested into acidic solution (e.g., mixture of 1.0 mL of 70% nitric acid, 1.0 mL of 36% Hydrochloric acid, 0.5 mL of 60% Hydrofluoric acid, and 4 mL of water). When the zeolite sample was completely dissolved into acidic solution, Hydrofluoric acid was neutralized with the addition of boric acid (e.g., 2.0 g). Furthermore, this digested mixture was further diluted with distilled water to obtain the desired concentration (1 ppm). BET analysis of catalyst samples was carried out using the Nitrogen sorption method (Micrometrics TriStar II 3020). Before BET analysis, all catalyst samples were degassed at 300 °C for 120 min for the removal of water amount from the catalyst sample. Nitrogen adsorption–desorption procedure was conducted for the measurement of porosity. The Brunauer–Emmett–Teller (BET) method was used to estimate specific surface area of solid catalyst samples [22]. Subsequently, the mesopore size distribution was calculated from the adsorption branch of the isotherm (BJH pore size model). Similarly, the micropore volume characteristic was measured using the t-plot method according to Lippens and de Boer.

Field emission scanning electron microscopy (FE-SEM, MIRA-3 Tescan, Hanyang University, Ansan, South Korea) and high-resolution transmission electron microscopy (HRTEM, Jeol JEM2100F, Hanyang University, Ansan, South Korea) were used to study the surface morphology of the hierarchical zeolite samples. A few droplets of the solid zeolitic sample suspended in ethanol were placed on a carbon-coated copper grid, followed by evaporation at ambient conditions. Similarly, SEM-EDX analysis was conducted. For SEM analysis, a platinum coating was used to make thin films. For HR-TEM analysis, the samples were dissolved in ethanol and deposited on TEM grit and then dried at 60 °C in oven for 2 days.

Temperature-programmed reduction (TPR) technique was used to characterize metal-supported zeolite samples chemically. These TPR-based experiments were performed in a Okhura TPD 20,025 unit under the use of 5% H_2_/Ar gaseous mixture at 60 cm^3^/min at STP conditions. TPR equipment was operated in temperature range of 60 to 800 °C at 10 °C/min rate.

The metal dispersion of Ni-supported catalysts was determined by the chemisorption of hydrogen using ASAP 2020C V1.09G (Micromeritics Inst. Co, available at KRICT Daejeon, Republic of Korea) in accordance with the published procedure [23].

Temperature-programmed desorption method was used to analyze the acidity strength of catalyst samples (Auto Chem 2920, Micrometrics, USA, available at KRICT, Daejeon, Republic of Korea). Ammonia gas was used for the adsorption process in the TPD system. Ammonia adsorption was conducted at a temperature of 50 °C at fixed flow rate (20 cm^3^ min^−1^) for 45 min. During this adsorption process, a helium purging step was followed at room temperature for the removal of physisorbed ammonia (desorption). The desorption process was recorded in the temperature range of 50−900 °C at a heating rate of 10 °C min^−1^ under a helium flow (20 cm^3^ min^−1^), and the evolved NH_3_ was monitored by TCD.

Infrared (IR) spectroscopic monitoring of adsorbed pyridine is an established tool for investigating the acidity of solid acid catalysts. The advantages of this technique are that Bronsted acid (B) and Lewis acid (L) sites can be distinguished. The acid strength distribution of sites could be investigated by monitoring the thermo-desorption of pyridine in the IR cell. The IR in situ cell consisted of a stainless steel housing onto which ZnSe windows were sealed. The cell contained a quartz-clad heating device in which a KBr pellet was inserted into a disk. The pump section consisted of a turbo-molecular pump connected to a rotary pump. Copper O rings were used in all flange connections. The designing of the IR cell was conducted for pyridine adsorption, as per a published paper procedure [22].

For acid site measurements, KBr-based pellets (15% catalyst amount added, 11 tons cm^−2^, 50 mg, 1 cm^2^) were dehydrated into especial designed stainless steel IR cells under vacuum (10^−3^ mbar) at 450 °C for 1 h and then cooled at 150 °C, and the IR spectrum of samples without pyridine adsorption was recorded (measured through FTIR apparatus (Nicolet, iS10 FTIR spectrometer) at conditions of 600–4000 cm^−1^, 8 cm^−1^ optical resolution, and co-addition of 32 scans). All measurements were performed at this temperature in order to prevent the physisorption of pyridine. After closing the valve between the cell and the pump system, pyridine gas was added stepwise in amounts of approximately 2 µmol. A spectrum was recorded after each 5 min after each addition. When no significant change was observed in the IR spectrum, then physisorbed pyridine was removed by applying heating at 350 °C for 15 min under vacuum conditions; then, it was cooled up to 150 °C, and spectrum was recorded in order to assess the amount of strongly acidic sites. The accurate IR spectrum of the material was collected after subtracting the spectrum of the dehydrated sample from the pyridine-adsorbed spectrum [24,25]. The quantitative number of acidic sites was calculated using the following formula of a previously published paper [35]:

C (pyridine on B sites): C(L) = 1.42 × IA(LA) × R^2^/Wcat (1)

C(pyridine on L sites): C(B) = 1.88 × IA(BA) × R^2^/Wcat (2)

where C(L) and C(B) show the concentrations of Lewis and Brønsted acid sites, respectively, and IA (L) and IA (B) indicate the integrated surface areas of Lewis and Brønsted acid site peaks (1440 and 1540 cm^−1^), respectively. R is the radius of the catalyst disk/pellet (cm), and Wcat is the total amount of catalyst (mg) that was used in the preparation of the KBr pellet. The integrated molecular extinction coefficients for Bronsted and Lewis bands are 1.67 and 2.22 cm/µmol, respectively. These IMEC values were calculated as per a published paper [35]. The above Equations (1) and (2) were obtained by using IMEC values and the following equation (Equation (3)):

3.14 R^2^ [slope (B)/IMEC (B) + Slope (L)/IMEC (L) = 1 (3)

### 3.4. One-Pot Synthesis of Menthols from Liquid-Phase Citral Hydrogenation

One-pot menthol synthesis reaction (e.g., citral hydrogenation reaction) was carried out in a stainless steel high-pressure autoclave hydrogenation reactor under optimized reaction conditions. The specific amounts of chemicals such as 6.5 mmol citral, 0.2 g freshly activated catalyst, 25 mL toluene solvent, and 0.2 mL nitrobenzene (standard reagent as internal GC standard) were added into the autoclave hydrogenation reactor and assembled properly with a heating mantle and stirrer for hydrogenation reaction. Before the reaction start, the reactor and sample lining were purged with hydrogen gas three times. Furthermore, the specific reaction temperature of 100 °C and hydrogen gas pressure of 1.0 MPa were maintained before the reaction start. The reaction was preceded at a constant stirring speed (300 rpm). The reaction samples were collected from the autoclave hydrogenation reactor for GC product analysis. For sample collection, syringe filters were used to remove dust impurities from samples. GC analysis of reaction samples was conducted using an Agilent gas chromatograph (chiral Cyclodex-B column: length, 60 m; diameter, 0.254 mm; and film thickness, 0.25 µm). Helium gas was used as the carrier gas. The products were separated by the following temperature program: 95 °C (1 min), 0.3 °C min^−1^, 120 °C (1 min), 15 °C min^−1^, and 220 °C (1 min), with a split ratio of 100:1. The detector and injector temperatures were 280 and 250 °C, respectively. The reaction products and isomers were further identified by GC–MS with a triple-axis detector (Agilent, model no. 7890A) and GC chemical standards. The GC temperature-programing operating conditions used were already mentioned in our previously published paper [6]. Further, detailed reaction product analyses were carried out through GC-MSD and GC grade chemicals using similar GC operating conditions, as discussed above.

**Scheme 3:**
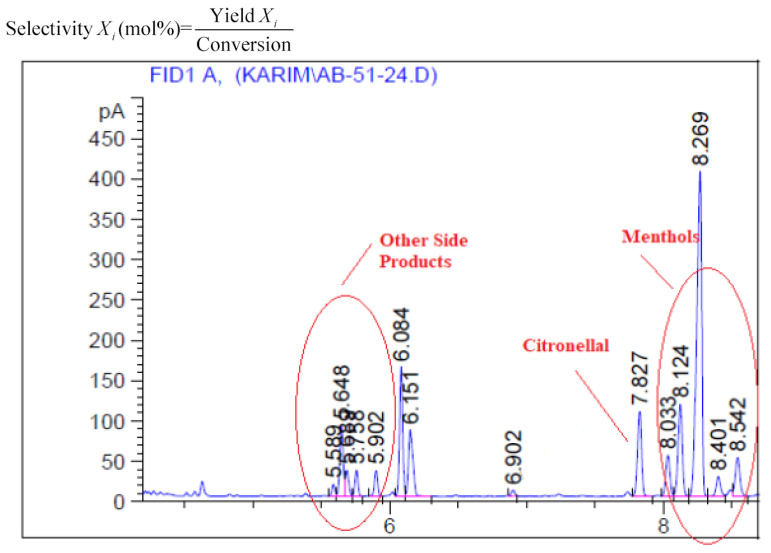
Gas chromatograph of citral hydrogenation to Menthol reaction.

Furthermore, reaction conversion and product selectivity calculations were made using gas chromatograph (Scheme 3) and formula.

## 4. Conclusions

In this research, the effects of alkali treatment on the textural, acidic, and structural properties of zeolites (ZSM-5 and USY) were investigated and correlated with the citral hydrogenation reaction performance. A number of hierarchical zeolites were prepared at various alkali concentrations using the desilication reassembly process. Afterwards, the Ni precursor was doped over hierarchical zeolites. The characterization of all catalysts, such as XRD, sorption, H_2_-TPR, NH_3_-TPD, and ICP-OES, was conducted, and their interconnection with catalytic activity and menthol synthesis was found. The XRD structure of zeolites was maintained at optimum alkali treatment (0.5 M NaOH), whereas the amount of mesoporosity and acidity was enhanced with alkali treatment. The number of Lewis acid sites were increased with alkali treatment. The catalytic activity or performance of purely microporous ZSM-5 was improved through the desilication reassembly process, which resulted in wide mesopore formation, as well as the enhancement in the mesopore surface area and acidity. Nevertheless, it was revealed that the improved physicochemical characteristics of zeolite have a positive impact on menthol production, as well as catalytic activity enhancement. For effective citronellal cyclisation, balanced metal acid sites (strong Lewis and weaker Bronsted acid sites) were maintained on the surface of the hierarchical zeolite exterior surface. The mass transfer and molecular diffusion rates were improved due to the formation of micro-mesoporous structures because of Si leaching and structural reshaping. A number of cracked products were formed using the USY80 catalyst; this cracking was seen because of high-strength Bronsted acidity. The cracking rate declined through the desilication of USY80 because of a reduction in Bronsted acidity and an increase in Lewis acidity. Finally, it can be concluded that the Ni-HZ-0.5 M catalyst (TOF 7.12 h^−1^) was the most active and selective catalyst for one-pot menthol synthesis because of improved mesoporosity, acidity, and metal dispersion, along with good recyclability.

## Figures and Tables

**Table 1:** Characterization data of Ni-supported hierarchical zeolites samples.

Samples	S_tot_^a^	S_meso_^c^	V_tot_^a^	V_micro_^b^	V_meso_^c^	ICP-OES ^d^	
	m^2^·g^−1^	m^2^·g^−1^	cm^3^·g^−1^	cm^3^·g^−1^	cm^3^·g^−1^	Ni (%)	Si/Al
Ni-Z50	308	43	0.249	0.15	0.1	6.93	50
Ni-HZ-0.2 M	355	180	0.34	0.15	0.25	6.96	28
Ni-HZ-0.5 M	377	300	0.54	0.13	0.5	7.06	25
Ni-HZ-0.7 M	395	301	0.819	0.05	0.78	7.3	29
Ni-USY80	655	260	0.47	0.3	0.27	5.9	80
Ni-HZ-USY80	679	730	0.57	0.16	0.64	5.98	45

Note: ^a^ S_BET_ and V_tot_ were calculated by applying the multi-point BET equation in linear range. ^b^ V_micro_ was calculated by applying the t-plot method. ^c^ S_meso_ and V_meso_ were calculated by BJH method. ^d^ was determined by ICP-OES technique.

**Table 2:** Chemisorption data of Ni-supported catalysts.

Sample	H_2_-TPR (C) ^a^		Ni Dispersion ^b^	Crystallite Size ^c^
	LT	HT	%	NiO (nm)
Ni/Z50	359	510	5.04	20
Ni/HZ-0.2 M	396	523	2.95	34
Ni/HZ-0.5 M	390	573	3.97	25
Ni/HZ-0.7 M	362	615	4.99	20
Ni/USY80	340	600	1.89	5.0
Ni/HZ-USY80	328	635	3.38	30

^a^ LT and HT profiles of zeolite samples, ^b^ measured through H_2_-Chemisoprtion technique, ^c^ measured through XRD technique.

**Table 3:** Acidic and elemental composition characteristics of hierarchical zeolite samples.

Catalyst Type	NH_3_-TPD Acidity (mmol) ^a^			Acidic Sites ^b^			Al ^c^
	Low	Medium	Total	L.A	B.A	B/L	µmol·g^−1^
Ni/Z50	0.84	0.42	1.25	198	268	1.35	133
Ni/HZ-0.2 M	0.89	0.63	1.51	293	99	0.34	140
Ni/HZ-0.5 M	1.1	0.44	1.53	428	163	0.38	199
Ni/HZ-0.7 M	1.53	0.59	2.12	430	118	0.36	270
Ni/USY80	1.78	0.64	2.42	78	284	3.66	n.d
Ni/HZ-USY80	1.85	0.69	2.54	167	152	0.311	n.d

Note: ^a^ total acidity strength was measured through NH_3_-TPD technique. ^b^ quantitative acid sites (L/B) conc. was measured via pyridine adsorption technique. ^c^ determined by ICP-OES technique.

**Table 4:** Catalytic activities of Ni-supported hierarchical zeolites catalysts in citral hydrogenation to menthols.

Catalyst Type	Menthols		Products Distributions					TOF ^a^
	Select. (%)	Yield (%)	Isopulegol	Citronellal	3,7 DMOL	Citronellol	Others	(h^−1^)
Ni-Z50 *	4	2.5	3.26	57.8	0	1.9	2.7	0.8
Ni-HZ-0.2 M	17	17.3	12.42	57.0	1.68	7.59	3.3	1.9
Ni-HZ-0.5 M	83	82.43	0	1.87	2.6	3	10.7	7.12
Ni-HZ-0.7 M	47	46.42	5.5	17.23	2.28	19.8	6.7	7.14
Ni-USY80	53	53.4	1.7	0	6.18	0	38.5	0.56
Ni-HZ-USY80	58	58.04	2.33	0	5.82	0	33.6	1.30

Note: Reaction conditions: 6.57 mmol citral, 25 mL toluene, 0.2 g freshly reduced catalyst, 100 °C, 1.0 MPa H_2,_ reaction time 4 h, and 100% conversion. * gave 34% conversion only. ^a^ TOF was calculated by total moles of citral * total conversion * MW of citral divided by 100 * catalyst weight * reaction time.

**Table 5:** Recyclability test of Ni/HZ-0.5 M catalyst in citral hydrogenation.

No. of Cycles	Conversion (%)	Selectivity (%)	Treatment
1	100	83	Freshly reduced
2	100	81	Calcined and reduced 500 °C-4 h
3	98	78	Calcined and reduced 500 °C-4 h

Note: reaction conditions—1.0 g citral, 25 mL toluene, 0.2 g freshly reduced catalyst, 100 °C, and 1.0 MPa H_2._

**Table 6:** Catalytic performances of different bifunctional catalysts in menthol synthesis.

Catalysts	Conversion (%)	Overall Selectivity (%)	References
15% Ni/Zr-beta-HDP	100	44	[31]
2% Pd-Zr-beta	100	62	[31]
Ni-H-MCM-41	100	54	[32]
Ni-H-Y	42	4	[32]
Pd-PTA-Al_2_O_3_	100	45	[33]
Pd-PMA-Al_2_O_3_	100	27	[33]
Pd-SMA-Al_2_O_3_	67	4	[33]
Pd-STA-Al_2_O_3_	78	13	[33]
Ni-PTA-MMT	100	41	[6]
Pd-MMT	100	35	[6]
5% Ni/MAS	100	75	[34]
5% Ni/H-beta-38	88	49	[34]
Ru/H-MCM-41	70	8	[34]
Ru/H-Y-80	42	6	[34]
Pd-30% PTA-ALPO	6	5.9	Our Study
Ni-Z50 * Ni-Dealuminated zeolite *	34 24	4.0 23.8	Our Study
Ni-HZ-0.2 M *	100	17	Our Study
Ni-HZ-0.5 M *	100	83	Our Study
Ni-HZ-0.7 M *	100	47	Our Study
Ni-USY80 *	100	53	Our Study
Ni-HZ-USY80 *	100	58	Our Study

Note: * 6.56 mmol, citral; 25 mL, C_6_H_5_CH_3_; 0.2 g, catalyst; T, 100 °C; a1.0 MPa, H_2_ gas; and reaction period, 2–16 h.

## Data Availability

Not applicable.
